# Oral Vaccination of Recombinant *Saccharomyces cerevisiae* Expressing ORF132 Induces Protective Immunity against Cyprinid Herpesvirus-2

**DOI:** 10.3390/vaccines11010186

**Published:** 2023-01-16

**Authors:** Licong Wang, Maoxia Yang, Sheng Luo, Guanjun Yang, Xinjiang Lu, Jianfei Lu, Jiong Chen

**Affiliations:** 1 State Key Laboratory for Managing Biotic and Chemical Threats to the Quality and Safety of Agro-Products, Ningbo University, Ningbo 315211, China; 2 Laboratory of Biochemistry and Molecular Biology, School of Marine Sciences, Ningbo University, Ningbo 315211, China; 3 Key Laboratory of Applied Marine Biotechnology of Ministry of Education, Ningbo University, Ningbo 315211, China

**Keywords:** crucian carp, CyHV-2, yeast surface display, oral vaccination, ORF132

## Abstract

Cyprinid herpesvirus 2 (CyHV-2) is the etiological agent of herpesviral hematopoietic necrosis (HVHN) disease, which causes serious economic losses in the crucian carp culture industry. In this study, by displaying ORF132 on the surface of *Saccharomyces cerevisiae* cells (named EBY100/pYD1-ORF132), we evaluated the protective efficacy of oral administration against CyHV-2 infection. Intense innate and adaptive immune responses were evoked in both mucosal and systemic tissues after oral vaccination with EBY100/pYD1-ORF132. Importantly, oral vaccination provided significant protection for crucian carp post CyHV-2 infection, resulting in a relative percent survival (RPS) of 64%. In addition, oral administration suppressed the virus load and relieved histological damage in selected tissues. Our results indicated that surface-displayed ORF132 on *S. cerevisiae* could be used as potential oral vaccine against CyHV-2 infection.

## 1. Introduction

Crucian carp (*Carassius auratus gibelio*), belonging to the genus *Carassius* within the family Cyprinidae, is a major freshwater aquaculture species worldwide [1]. However, herpesviral hematopoietic necrosis (HVHN) caused by Cyprinid Herpesvirus-2 (CyHV-2) leads to acute mass mortality in populations of crucian carp, which seriously harms the development of crucian carp culture industry [2]. CyHV-2, a member of the genus *Cyprinivirus* within the family Alloherpesviridae, has a lipid envelope bearing viral glycoproteins and a 290.3 kb linear double-stranded DNA genome [3,4]. The typical clinical symptoms of this disease are kidney and spleen enlargement, pale gills, eye protrusion, body surface congestion, and abdominal swelling [5]. Since it was first detected in goldfish (*Carassius auratus*) in Japan, the disease caused by CyHV-2 has been reported worldwide [6,7]. Thus, there is an urgent need to adopt effective strategies to control HVHN and minimize the consecutive losses in aquaculture.

Vaccines play an important role in the prevention and control of infectious diseases in aquaculture [8]. It is reported that fish vaccines include live, inactivated, and subunit vaccines, and the methods of administration include immersion, injection, and oral routes [9]. Immersion and injection were common immunization routes, but neither was the optimal delivery method for large-scale administration in aquaculture industry [10]. The disadvantages of immunization by injection are high labor cost, complex operation, and harm to the fish during the process. In addition, the protective efficacy of immersion was not only greatly affected by the immersion environment, but it was also lower than injection immunization. On the contrary, oral immunization was considered an appropriate method for immunizing large numbers of farmed fish at all life stages, due to its limited stress for fish, fewer labor requirements, easy application, and safety [10].

Yeast is an ideal carrier for vaccine production, which has the advantages of adjuvant function, easy culture, low production cost, and safety. The yeast surface display technology displays foreign protein by using a yeast a-agglutinin receptor, which is particularly useful in protein engineering, antibody development, and vaccines [11]. Because of these advantages, the yeast surface display system is widely used in vaccine development, especially oral vaccines. For instance, yeast surface display technology was applied to develop an oral vaccine against severe acute respiratory syndrome coronavirus 2 (SARS-CoV-2) [12]. Yeast surface display technology was also successfully applied to prepare an oral vaccine against lethal H_7_N_9_ avian influenza challenge in mice [11]. On the other hand, yeast display technology is widely used in fish vaccines. An oral vaccine displaying the infectious hematopoietic necrosis virus (IHNV) G protein on yeast cells could induce immune responses and protect rainbow trout from IHNV infection [13]. In another report, oral pORF65 vaccine based on the yeast surface display technology offered immune protection against cyprinid herpesvirus-3 (CyHV-3) infection [14]. Additionally, VP7 of grass carp reovirus (GCRV) displayed on the surface of *Saccharomyces cerevisiae* might be a dominating antigenic protein, which could be used to develop an efficient vaccine against GCRV [15]. However, yeast vaccines against CyHV-2 infection have been rarely studied.

ORF132 is an envelope protein of CyHV-2 which has been explored in the previous study [16]. Through antigenic prediction, we found that ORF132 was enriched in antigen determining clusters. We developed an oral vaccine (named EBY100/pYD1-ORF132) in which ORF132 was displayed on the *S. cerevisiae* surface. Oral vaccination with EBY100/pYD1-ORF132 could induce a significant immune response, protect crucian carp against CyHV-2 infection, and decrease virus load in CyHV-2-infected crucian carp. Hence, oral administration of EBY100/pYD1-ORF132 could be an attractive CyHV-2 vaccine candidate for further investigation.

## 2. Material and Methods

### 2.1. Fish and Virus

Crucian carp weighing 15–18 g and length 9–10 cm were purchased from a fishery in Chunxiao County, Ningbo City, China. Fish were acclimatized in a recirculating aquaculture system with freshwater (20–23 °C) for 14 days. The fish were fed with commercial fodder twice a day. Before the experiment, fish were randomly sampled for CyHV-2 detection by PCR, and CyHV-2 antibody was detected in fish serum via ELISA.

The CyHV-2 strain used in this study was isolated in our previous study [17].

### 2.2. Construction of Recombinant pYD1-ORF132

Antigenic epitope of ORF132 (protein ID: AMB21701.1) was analyzed by ABCpred (http://www.cbs.dtu.dk/services/BepiPred/) (accessed on 1 December 2021) and DNAStar (https://www.dnastar.com/ (accessed on 1 December 2021)). An antigenic epitope fragment rich region (amino acids 26–152) was selected for further investigation. Total DNA of CyHV-2 infected tissues was extracted using Tissue Genomic DNA Isolation Kit (Tiangen, Beijing, China) according to the manufacturer’ instructions. The amino acids 26–152 of ORF132 were amplified with gene-specific primers containing *BamH I* and *EcoR I* sites (Table 1). Then, the objective product was inserted into pYD1 vector and the recombinant plasmid screened by PCR and sequencing. The recombinant plasmid was named pYD1-ORF132.

### 2.3. Construction of Recombinant EBY100/pYD1-ORF132

To construct a *S. cerevisiae* (EBY100) carrying the recombinant vector pYD1-ORF132, recombinant plasmid was transformed into EBY100 competent cells according to the instruction manual of Super Yeast Transformation Kit (Coolaber, Beijing, China). EBY100 containing pYD1-ORF132 vector was denoted EBY100/pYD1-ORF132, and EBY100 containing pYD1 vector was denoted EBY100/pYD1. The recombinant pYD1-ORF132 protein was induced to express ORF132 by using yeast nitrogen base-casamino acid (YNB-CAA) medium containing 2% galactose (0.67% YNB, 0.5% CAA, 2% glucose). The presence of ORF132 protein was detected via immunofluorescence assay.

### 2.4. Immunofluorescence Assay

EBY100/pYD1-ORF132 cells were inducted with 2% galactose for 72 h. Cells were collected and washed three times with PBS. Followed by inoculation with monoclonal mouse anti-His antibody (1:500 diluted) (BBI, Shanghai, China) for 1 h at room temperature, the cells were treated with Alexa Fluor 555-labeled Donkey Anti-Mouse IgG (1:500 diluted) (Beyotime, Hangzhou, China). Finally, EBY100/pYD1-ORF132 cells were collected and photographed using a confocal laser scanning microscope LSM880 (Carl Zeiss, Jena, Germany).

### 2.5. Western Blotting

Cells were resolved by 12% SDS-PAGE and transferred into 0.45 μm PVDF membrane (Merck Millipore, Darmstadt, Germany) electrophoretically. Membrane was blocked with 5% nonfat milk at room temperature for 2 h, followed by incubation with a monoclonal mouse anti-His antibody (1:5000 diluted) (BBI) at 4 °C overnight. Subsequently, the membrane was incubated with HRP conjugated goat anti-mouse lgG (1:5000 diluted) (BBI) at room temperature for 2 h, after being washed 3 times with PBST (0.1% Tween 20). Finally, the protein band was captured by TanonImage software (Tanon, Shanghai, China) with ECL Western blot analysis kit (Beyotime).

### 2.6. Oral Immunization and Experimental Design

Six hundred crucian carp were divided into three groups (each group contains three parallel groups) and oral gavage with 0.1 mL PBS, or 2 × 10^9^ yeast cell (EBY100/pYD1 or EBY100/pYD1-ORF132) supplemented in 0.1 mL PBS, respectively. The oral immunization was conducted once a day for one week. Seven days later, oral immunization was repeated for another week. Before sampling, fish were euthanized using anesthetic (0.03% [*v*/*v*] ethylene glycol monophenyl ether). The blood was collected by venipuncture on day 7, 14, 21, and 28 post last immunization, and centrifuged at 5000× *g* for 15 min, then the obtained serum was stored at −80 °C until use. Meanwhile, fish hindgut, spleen, and head–kidney were sampled (9 fish each) on day 7, 14, 21, and 28, and stored at −80 °C until use.

For the CyHV-2 challenge experiment, 450 fish divided into three groups (each group contains three parallel groups of 50 fish each) received intraperitoneal injection with CyHV-2 suspension at a dose of 0.1 mL 1 × 10^6^ TCID_50_/g CyHV-2 [18]. Fish mortality in each group was recorded for 15 days. The RPS was calculated by: RPS% = (mortality of control group% − mortality of immunized group%)/mortality of control group%.

### 2.7. RNA Extraction, cDNA Synthesis, and Quantitative Real-Time PCR (qPCR)

The expression of several immune-related genes was assessed for immune response evaluation, including *immunoglobulin M* (*IgM*), *immunoglobulin T* (*IgT*), *interferon 1* (*IFN-1*), *tumor necrosis factor-α* (*TNF-α*), and *interleukin-1β* (*IL-1β*). House-keeping gene *18s rRNA* served as the internal control. All qPCR primers were shown in Table 1. The qPCR assay was performed as outlined in our previously described study [19]. Briefly, total RNA extraction of fish hindgut, spleen, and head–kidney were extracted by TRIzol Reagent (Takara, Dalian, China) according to the manufacturer’s description. The qPCR was performed using TB Green Premix Ex Taq™ (Takara) in an ABI QuantStudio 3 Real-Time PCR System (ABI, Foster City, CA, USA). The 2^−ΔΔCT^ method was used to calculate the relative expression levels of the different genes [20]. Data were presented as gene expression level normalized with *18s rRNA* or fold change in comparison to the corresponding control group.

### 2.8. Detection of ORF132 Specific Antibody

ELISA was used to analyze ORF132 specific antibody induced by EBY100/pYD1-ORF132 in crucian carp serum. The ELISA plate was coated with 1 µg/mL fish serum and blocked with PBS containing 5% skimmed milk (Sangon, Shanghai, China) for 2 h at 37 °C. After that, the plates were incubated with 1 µg ORF132 amino acid residue (KKKKKTTKKSSSKSSGKNKRSGGYDN) for 1 h at 37 °C. ORF132 polyclonal antibody from mouse was custom made in GL Biochem (Shanghai, China) using amino acid residue (KKKKKTTKKSSSKSSGKNKRSGGYDN). Next, 100 µL ORF132 polyclonal antibody (1:2000 diluted) was added to each well and incubated 1 h at 37 °C. Next, HRP-conjugated Goat Anti-mouse IgG (1:5000 diluted) (Beyotime) was used to recognize ORF132 polyclonal antibody. Finally, TMB (TianGen) was used as colorimetric substrate and the plates were read at 450 nm with a microplate reader (Potenov, Beijing, China).

ORF132 was analyzed by ABCpred and DNAStar, antigenic epitope fragment rich region (amino acids 127–152, KKKKKTTKKSSSKSSGKNKRSGGYDN) was selected to produce ORF132 polyclonal antibody. ORF132 polyclonal antibody was custom made in GL Biochem Ltd. (Shanghai, China) by peptides amino acids 127–152.

### 2.9. Histological Examination

Histopathological examination was performed as in our previous study [21]. Three fish from each group were randomly selected at 48 h post infection. Head–kidney, spleen, and hindgut of selected fish were collected for histological examination. Samples were dissected and washed with saline three times and fixed in Bouin’s solution for 24 h. Before being embedded in paraffin wax, samples were dehydrated by gradient ethanol. Paraffin sample sections (4 μm thickness) were stained with hematoxylin and eosin and examined using a light microscope (Olympus, Tokyo, Japan).

### 2.10. Statistics Analysis

Data were analyzed by one-way analysis of variance (ANOVA) and Duncan’s multiple range test. Results are presented as the mean ± standard error of the mean (SEM). *p* < 0.05 were considered statistically significant.

## 3. Results

### 3.1. Construction of the Recombinant S. cerevisiae Strain Expressing ORF132

**Figure 1:**
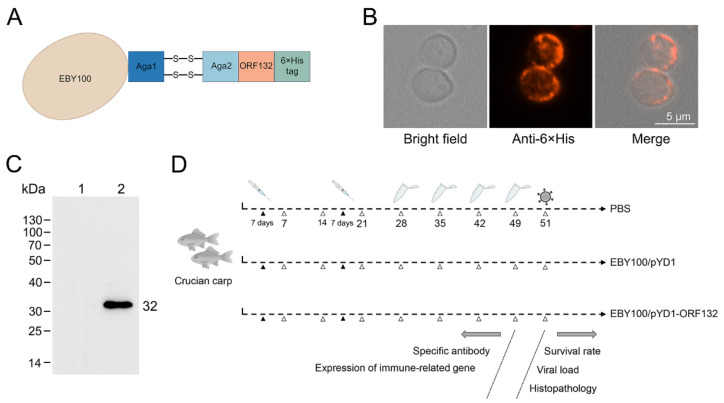
The construction and characterization of ORF132 on the EBY100 surface. (**A**) Schematic diagram of recombinant yeast vaccine construction. The epitope antigen of ORF132 was displayed on the surface of cell wall by Aga1 and Aga2. (**B**) ORF132 expressed on the surface of EBY100 yeast cells were testified using confocal microscopy. bar = 5 μm. (**C**) ORF132 protein was determined by Western blot. 1, EBY100/pYD1; 2, EBY100/pYD1-ORF132. (**D**) Schematic illustration of vaccine immunization for crucian carp.

*S. cerevisiae* (EBY100) was used as the vaccine platform to develop a novel CyHV-2 vaccine. The ORF132 epitope antigen was inserted between Aga2 and 6× His tag, which enabled ORF132 epitope antigen to be presented on the surface of *S. cerevisiae* (Figure 1A). Mouse anti-His antibody was used to detect ORF132 derived in EBY100. Fluorescence microscopy showed that red fluorescence was visible on the yeast cell surface, which indicted that ORF132 was displayed on the surface of the yeast cells (Figure 1B). Western blot assay showed that ORF132 protein could be recognized by anti-His antibody. The experimental procedure of this study is shown in Figure 1C.

### 3.2. Specific Antibody Induced by Oral Vaccination

**Figure 2:**
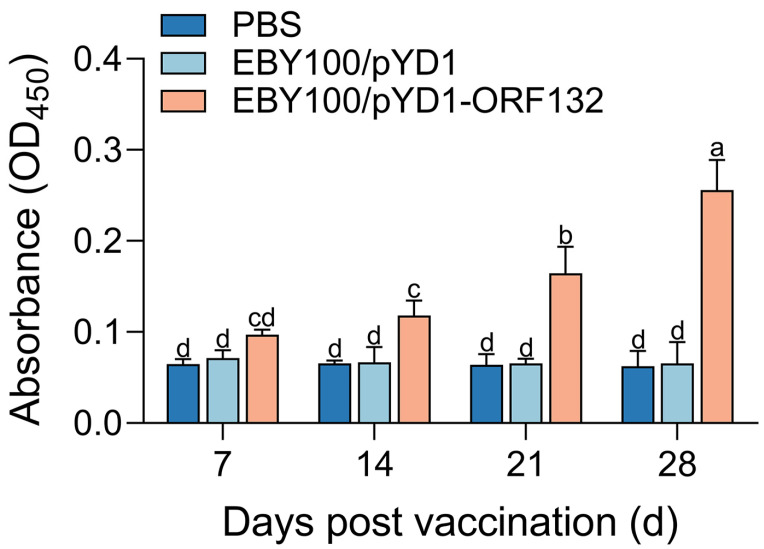
ORF132 specific antibody induced by oral vaccination. Fish serum from crucian carp was collected on day 7, 14, 21, and 28, and the antigen-specific immune responses were analyzed by ELISA. Bars with different letters were significantly different according to the Duncan’s multiple range (*p* < 0.05).

ORF132 specific antibody induced by oral vaccination was detected by ELISA. As shown in Figure 2, the antibody levels of the EBY100/pYD1-ORF132 immunized group were significantly higher than that of EBY100/pYD1 or PBS immunized group (*p* < 0.05). EBY100/pYD1-ORF132 immunized group antibody appeared to show an upward trend over time, while the other two groups remained stable. On day 28, the highest antibody level was detected in the EBY100/pYD1-ORF132 immunized group.

### 3.3. The Expression of Immune-Related Genes after Vaccination

**Figure 3:**
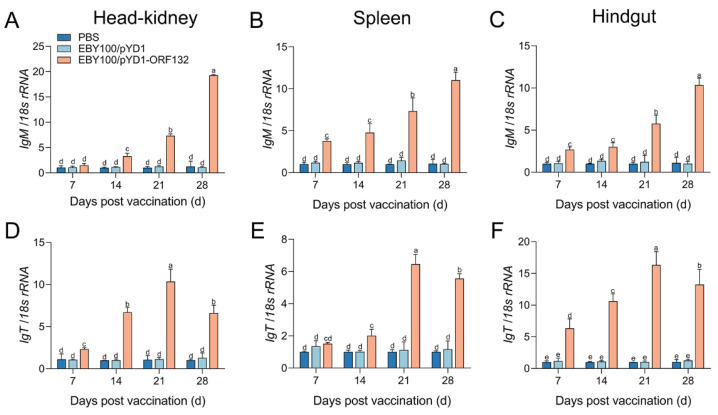
mRNA expression levels of *IgM* and *IgT* in the head–kidney (**A**,**D**), spleen (**B**,**E**), and hindgut (**C**,**F**) after the oral vaccination. Bars with different letters were significantly different according to Duncan’s multiple range (*p* < 0.05).

**Figure 4:**
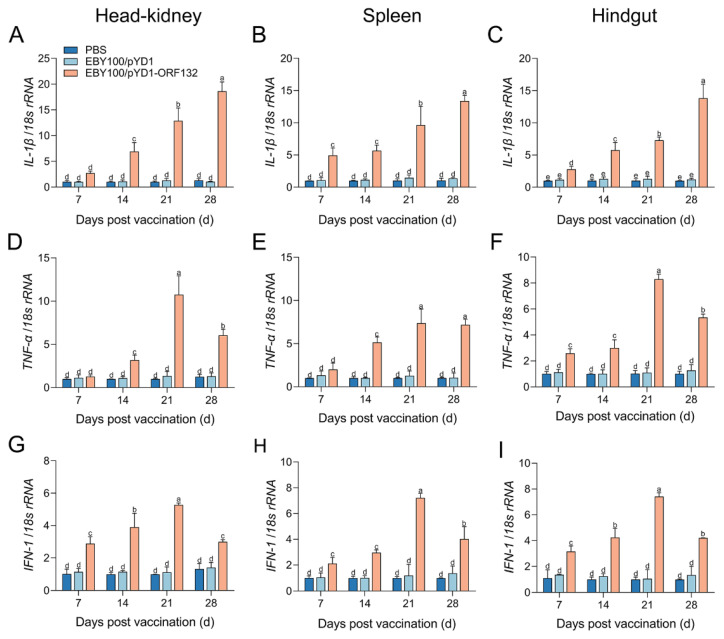
mRNA expression levels of *IFN-1*, *TNF-α,* and *IL-1β* in the head–kidney (**A**,**D**,**G**), spleen (**B**,**E**,**H**), and hindgut (**C**,**F**,**I**) after the oral vaccination. Bars with different letters were significantly different according to Duncan’s multiple range (*p* < 0.05).

The distributions of immune-related genes (*IgM*, *IgT*, *IFN-1*, *TNF-α,* and *IL-1β*) expression levels were analyzed by qPCR in the crucian carp systemic (head–kidney and spleen) and mucosal (hindgut) tissues. The result revealed that all the detected immune-related genes were upregulated significantly after EBY100/pYD1-ORF132 exposure (Figure 3 and Figure 4).

Immunoglobulins (Igs) are a crucial component of adaptive immunity [22]. In this study, we demonstrated that the *IgM* and *IgT* mRNA expression levels were significantly increased in the head–kidney, spleen, and hindgut after vaccination. In the hindgut, the maximum expression level of *IgT* was higher than that in the head–kidney and spleen after vaccination. Moreover, this higher expression started earlier in the hindgut (to 6.35-fold in 7 d) than the head–kidney (to 2.33-fold in 7 d) and spleen (to 1.52-fold in 7 d). On the contrary, the expression levels of *IgM* and *IgT* both increased notably in the systemic tissues, in which *IgM* increased more than *IgT*, and the increases in *IgM* expression level were dominant (Figure 3).

We also examined the distributions of *IFN-1*, *TNF-α,* and *IL-1β* mRNA levels in the crucian carp head–kidney, spleen, and hindgut post oral vaccination. Compared with EBY100/pYD1 or PBS immunized group, the administration of EBY100/pYD1-ORF132 induced a strong increase in the expression of *IL-1β*, *TNF-α*, and *IFN-1*. In addition, these immune-related genes were significantly upregulated in a time-dependent manner (Figure 4).

### 3.4. Protective Effect of Oral Vaccine on CyHV-2-Infected Crucian Carp

**Figure 5:**
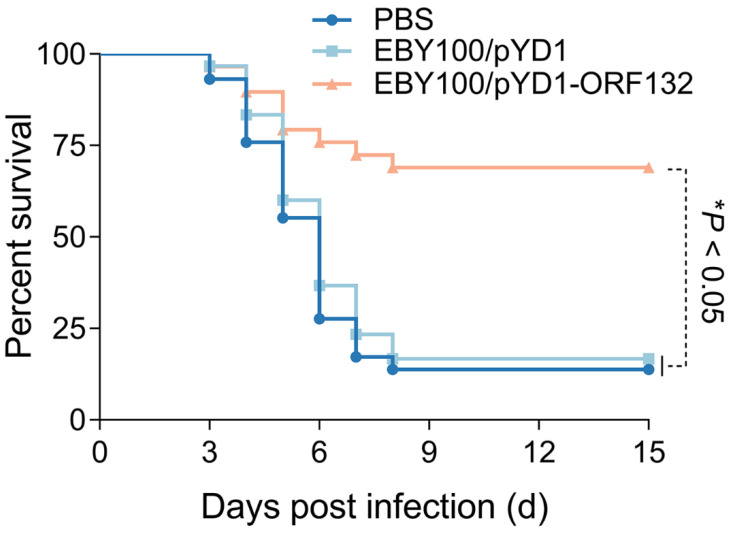
The cumulative mortality of vaccinated crucian carp. Mortality was recorded daily and analyzed based on the Kaplan–Meier survival curves, * *p* < 0.05.

Next, we investigated the mortality of crucian carp within 15 days post CyHV-2 challenge. Almost all dead fish showed the typical clinical symptoms, including eye protrusion, body surface congestion, and abdominal swelling. As shown in Figure 5, most of the fish in the PBS and EBY100/pYD1 group died within 6 days post infection; the remaining fish died within 9 days post infection. Importantly, upon challenge with CyHV-2, the EBY100/pYD1-ORF132 immunized group had a significantly higher survival rate (80%) compared with the PBS (16.7%) and EBY100/pYD1 (17.7%) groups. Therefore, the RPS in the EBY100/pYD1-ORF132 group was 64%. The results suggested that immunization formulation with EBY100/pYD1-ORF132 provided a certain amount of protection for crucian carp.

### 3.5. Oral Vaccine Decreased Virus Load in CyHV-2-Infected Crucian Carp

**Figure 6:**
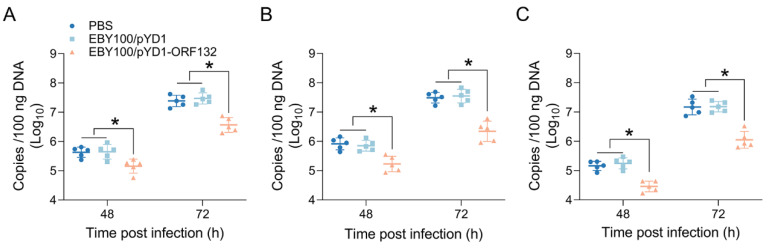
The virus load in head–kidney (**A**), spleen (**B**), and hindgut (**C**) at 48 and 72 h post CyHV-2 infection. The data were conducted using one-way analysis of variance with Bonferroni correction, and expressed as the mean ± SEM, *n* = 5, * *p* < 0.05.

To further examine the influence of oral vaccine on virus load, we analyzed the copy of CyHV-2 in the head–kidney, spleen, and hindgut post infection. As shown in Figure 6, CyHV-2 was detected in all selected tissues, and the virus load in the hindgut was lower than that in the head–kidney and spleen. In addition, the virus load increased with the infection time. Compared with the PBS and EBY100/pYD1 groups, the CyHV-2 load was significantly suppressed in the EBY100/pYD1-ORF132 group. The results suggested that oral immunization with EBY100/pYD1-ORF132 could effectively suppress the replication of CyHV-2.

### 3.6. Effect of Oral Vaccine on the Histopathology of CyHV-2-Infected Crucian Carp

**Figure 7:**
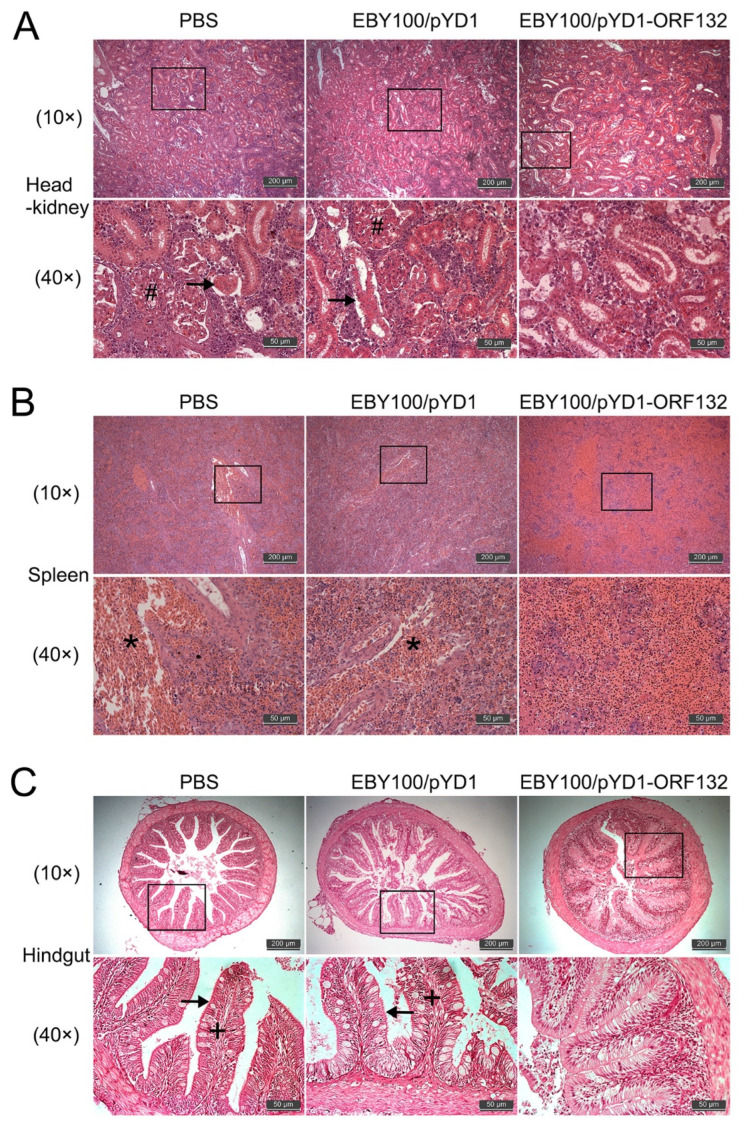
Histopathology analyses of head–kidney, spleen, and hindgut. Head–kidney (**A**), spleen (**B**), and hindgut (**C**) was performed for histopathological analysis. Organize shrinking (arrow), necrosis (#), diffuse congestion (*), and increase in goblet cells (+). Scale bar = 50 μm or 200 μm.

Histopathological examination was utilized to explore the effect of an oral vaccine on the histopathology of the tissues in CyHV-2-infected fish. Head–kidney, spleen, and hindgut samples were collected for histological analysis 2 days post CyHV-2 infection. In the head–kidney of PBS and EBY100/pYD1 groups, the renal tubular epithelia were separated from the basal lamina. Necrotic foci and organized shrinking were observed in tubular epithelial cells (Figure 7A). Additionally, diffuse congestion was observed in the spleen of PBS and EBY100/pYD1 groups (Figure 7B). However, no serious damage or minor lesions were observed in the EBY100/pYD1-ORF132 group. In the hindgut of PBS and EBY100/pYD1 groups, villi atrophy and an abnormal increase in goblet cells was observed (Figure 7C). Therefore, our results indicated that vaccination with EBY100/pYD1-ORF132 relieves the tissue damage caused by CyHV-2 infection.

## 4. Discussion

The epidemic of HVHN caused by CyHV-2 led to great economic losses in the aquaculture industry. However, there is no commercially available vaccine to prevent the disease. Yeast is considered an ideal carrier for vaccine production due to its advantages of adjuvant function, ease of culture, low production cost, and safety. Even compared with other biocarriers, such as *Lactobacillus* and *Bacillus subtilis*, using yeast as a vaccine has two outstanding advantages: (1) some of the cell components of yeast, such as β-glucan, mannan, and chitin, are known to have adjuvant potential; (2) yeast produces correct folds and is capable of performing post-translational modifications [11]. Here, using yeast display technology, we developed an oral vaccine with *S. cerevisiae* expressing CyHV-2 ORF132, which provided effective protection against CyHV-2 infection in crucian carp.

Neutralizing antibody plays a critical role in defending organisms against pathogens [23,24]. It was reported that oral delivery of recombinant *S. cerevisiae* cells could be engulfed by the intestinal epithelial cells of gut in crucian carp, and heterologous protein-specific antibodies could be detected in the serum of vaccinated fish [25]. This immune response could be rapidly upregulated after booster vaccination [26]. Results from the present study showed that ORF132 specific antibody in serum was induced after oral administration, and antibody levels in the EBY100/pYD1-ORF132 immunized group were significantly higher than that of the control group. The antigenic epitope of ORF32 was recognized by fish and stimulated specific serum antibody in serum. Thus, EBY100/pYD1-ORF132 was used in subsequent studies against CyHV-2 infection.

Oral administration is an ideal immunization route for fish at all life stages, especially the juvenile stage [10,27]. Displaying antigenic proteins on the surface of *S. cerevisiae* cells is a promising approach for the development of oral vaccines [24]. In this study, oral administration of EBY100/pYD1-ORF132 gave a 64% RPS in CyHV-2-infected crucian carp. Furthermore, oral administration of EBY100/pYD1-ORF132 was found to decrease virus load and histological damage in CyHV-2-infected fish. Moreover, vaccination with EBY100/pYD1-ORF132 relieves the tissue damage caused by CyHV-2 infection. The relief of tissue damage and lower virus load in vaccinated fish coincided with an improvement in the survival rate. The oral yeast vaccine needs to overcome the harsh intestinal environment, bringing about relatively low protection [27]. Previous studies have shown varying RPS in yeast surface display technology-based vaccines, including 53.3% [28] and 30% [14] for CyHV-3, 14.3% (one immunization) and 66.7% (two immunizations) for CyHV-2 [26], and 45.8% for IHHNV [13]. Unlike their experiment, two immunizations were performed in this study, each lasting 7 days. Thus, our experiment achieved a relatively high RPS (64%). Studies have shown that primary oral vaccination induces a weaker immune response, whereas intensive oral vaccination induces a strong secondary immune response and significantly improves fish survival [29]. The potential risk of oral yeast vaccination is dependent on the dose of antigen used and the route of administration, so the relationship between multiple dosing and fish survival should be further studied.

Vaccines evoke adaptive immunity and innate immunity in exposing the fish body to antigens [27]. We further conducted the expression of immune-related genes in vaccinated fish to evaluate the immune response of the prepared vaccine. The results in present study showed that EBY100/pYD1-ORF132 induced a significantly greater expression level of immune-related genes, including *IgM*, *IgT*, *IFN-1*, *TNF-α,* and *IL-1β*. Igs are a crucial component of adaptive immunity and three types Igs (IgM, IgD, and IgT/Z) have been described in teleost fish [22]. IgM is the main serum isotype in fish serum, while IgT is involved in mucosal protection [30,31]. In rainbow trout vaccinated with an oral vaccine against IHNV, *IgT* and *IgM* mRNA level was found to be increased in systemic and mucosal tissues, while *IgT* expression levels in the hindgut were higher than those in systemic tissues [13]. A similar phenomenon has also been found in crucian carp vaccinated with an oral yeast vaccine [26]. Here, *IgM* and *IgT* expression levels were promoted in the kidney, spleen, and hindgut post vaccination. However, the *IgT* expression level in the hindgut was much higher than that in systemic tissues. IFN-1 are cytokines produced by many cell types in response to viral infection [32,33]. TNF-α and IL-1β are important pro-inflammatory cytokines which mediate the production of inflammatory mediators [34]. In the present study, qRT-PCR results showed that oral vaccination with EBY100/pYD1-ORF132 induced significantly increased expression of immune-related genes, including *IFN-1*, *TNF-α,* and *IL-1β*. Overall, the results indicated that oral vaccination with EBY100/pYD1-ORF132 could elicit innate and adaptive immune responses in both mucosal and systemic tissues in crucian carp.

## 5. Conclusions

In summary, our report presents a preliminary study of an orally administered *S. cerevisiae* vaccine against CyHV-2 in crucian carp. The oral vaccine exhibited strong adaptive and innate immune responses in both mucosal and systemic tissues and improved the survival rate of CyHV-2-infected crucian carp. Furthermore, the histological damage in the head–kidney, spleen, and hindgut was remarkably decreased by the vaccine. The results indicated that the oral vaccine developed in this study is a promising candidate vaccine for controlling CyHV-2 infection in crucian carp farming.

## Figures and Tables

**Table 1:** Oligonucleotide primers used in this study.

Primer	Gene	Accession Number	Nucleotide Sequence (5′-3′)	Application
ORF132-F	*ORF132*	KT387800.1	CGCGGATCCGCTGCCACCCTGGGGACAG ^a^	pYD1 clone
ORF132-R			CCGGAATTCGGTATTGTCGTAGCCGC ^b^	
18s-F	*18s rRNA*	XR003291850.1	GAATGTCTGCCCTATCAACT	RT-qPCR
18s-R			GATGTGGTAGCCGTTTCT	
IgM-F	*IgM*	GU563726.1	GTCAATCTTCGGCTTGTCTCA	RT-qPCR
IgM-R			GGGTATAATCTCCATCGGGTC	
IgT-F	*IgT*	GQ201446.1	ATTCATTGTCAGACCTTCACTCAG	RT-qPCR
IgT-R			GCAGTGTCTTCAGTCTTGAGGCT	
IL-1β-F	*IL-1β*	AB757758.1	TTTGTGAAGATGCGCTGCTC	RT-qPCR
IL-1β-R			CCAATCTCGACCTTCCTGGTG	
TNF-α-F	*TNF-α*	KF500408.1	CGCTACTCTGATTCCTATGGC	RT10-qPCR
TNF-α-R			GCTTTCGCTGTTGCCTTTCT	
IFN-1-F	*IFN-1*	XM026209933.1	GTGGACATCCAGGGACTGTTAAG	RT-qPCR
IFN-1-R			CATTGCTCTGGATTCATGTGT	
CyHV-2-F	*ORF71*	KT387800.1	TTAGCGTCAGGTCCATAG	virus load
CyHV-2-R			GGCGTGTAGAAATCAAACT	

^a^ The underlined nucleotides represent the restriction site for *BamH I.* ^b^ The underlined nucleotides represent the restriction site for *EcoR I.*

## Data Availability

The data for this study are available in this article.

## References

[1] Davison A.J., Kurobe T., Gatherer D., Cunningham C., Korf I., Fukuda H., Hedrick R.P., Waltzek T.B. (2013). Comparative genomics of carp herpesviruses. J. Virol..

[2] Thangaraj R.S., Nithianantham S.R., Dharmaratnam A., Kumar R., Pradhan P.K., Thangalazhy Gopakumar S., Sood N. (2021). Cyprinid herpesvirus-2 (CyHV-2): A comprehensive review. Rev. Aquac..

[3] Lu J.F., Jin T.C., Zhou T., Lu X.J., Chen J.P., Chen J. (2021). Identification and characterization of a tumor necrosis factor receptor like protein encoded by Cyprinid herpesvirus-2. Dev. Comp. Immunol..

[4] Lu J.F., Shen Z.Y., Lu L.Q., Xu D. (2019). Cyprinid herpesvirus-2 miR-C12 Attenuates Virus-Mediated Apoptosis and Promotes Virus Propagation by Targeting Caspase 8. Front. Microbiol..

[5] Jeffery K.R., Bateman K., Bayley A., Feist S.W., Hulland J., Longshaw C., Stone D., Woolford G., Way K. (2007). Isolation of a Cyprinid herpesvirus-2 from goldfish, *Carassius auratus* (L.), in the UK. J. Fish Dis..

[6] Jung S.J., Miyazaki T. (1995). Herpesviral haematopoietic necrosis of goldfish, *Carassius auratus* (L.). J. Fish Dis..

[7] Wen J.X., Xu Y., Su M.Z., Lu L.Q., Wang H. (2021). Susceptibility of goldfish to Cyprinid herpesvirus-2 (CyHV-2) SH01 isolated from cultured crucian carp. Viruses.

[8] Barnes A.C., Silayeva O., Landos M., Dong H.T., Lusiastuti A., Phuoc L.H., Delamare-Deboutteville J. (2022). Autogenous vaccination in aquaculture: A locally enabled solution towards reduction of the global antimicrobial resistance problem. Rev. Aquac..

[9] Mondal H., Thomas J. (2022). A review on the recent advances and application of vaccines against fish pathogens in aquaculture. Aquac. Int..

[10] Angulo C., Tello-Olea M., Reyes-Becerril M., Monreal-Escalante E., Hernandez-Adame L., Angulo M., Mazon-Suastegui J.M. (2021). Developing oral nanovaccines for fish: A modern trend to fight infectious diseases. Rev. Aquac..

[11] Kumar R., Kumar P. (2019). Yeast-based vaccines: New perspective in vaccine development and application. FEMS. Yeast. Res..

[12] Gao T., Ren Y., Li S.Q., Lu X., Lei H. (2021). Immune response induced by oral administration with a *Saccharomyces cerevisiae*-based SARS-CoV-2 vaccine in mice. Microb. Cell Fact..

[13] Zhao J.Z., Xu L.M., Liu M., Cao Y.S., LaPatra S.E., Yin J.S., Liu H.B., Lu T.Y. (2017). Preliminary study of an oral vaccine against infectious hematopoietic necrosis virus using improved yeast surface display technology. Mol. Immunol..

[14] Ma Y.P., Liu Z.X., Hao L., Wu J., Qin B.T., Liang Z.L., Ma J.Y., Ke H., Yang H.W., Li Y.G. (2020). Oral vaccination using Artemia coated with recombinant *Saccharomyces cerevisiae* expressing Cyprinid herpesvirus-3 envelope antigen induces protective immunity in common carp (*Cyprinus carpio* var. Jian) larvae. Res. Vet. Sci..

[15] Luo S.X., Yan L.M., Zhang X.H., Yuan L., Fang Q., Zhang Y.A., Dai H.P. (2015). Yeast surface display of capsid protein VP7 of grass carp reovirus: Fundamental investigation for the development of vaccine against hemorrhagic disease. J. Microbiol. Biotechnol..

[16] Gao W., Wen H., Wang H., Lu J.Q., Lu L.Q., Jiang Y.S. (2020). Identification of structure proteins of Cyprinid herpesvirus-2. Aquaculture.

[17] Xu L.J., Podok P., Xie J., Lu L.Q. (2014). Comparative analysis of differential gene expression in kidney tissues of moribund and surviving crucian carp (*Carassius auratus gibelio*) in response to Cyprinid herpesvirus-2 infection. Arch. Virol..

[18] Lu J.F., Xu D., Jiang Y.S., Kong S.Y., Shen Z.Y., Xia S.Y., Lu L.Q. (2017). Integrated analysis of mRNA and viral miRNAs in the kidney of *Carassius auratus gibelio* response to Cyprinid herpesvirus-2. Sci. Rep..

[19] Luo S., Wang L.C., Shuai Z.H., Yang G.J., Lu J.F., Chen J. (2022). A short peptidoglycan recognition protein protects *Boleophthalmus pectinirostris* against bacterial infection via inhibiting bacterial activity. Fish Shellfish Immunol..

[20] Zhou Y., Qiu T.X., Hu Y., Liu L., Chen J. (2022). Antiviral effects of natural small molecules on aquatic rhabdovirus by interfering with early viral replication. Zool. Res..

[21] Wu X.Y., Xiong J.B., Fei C.J., Dai T., Zhu T.F., Zhao Z.Y., Pan J., Nie L., Chen J. (2022). Prior exposure to ciprofloxacin disrupts intestinal homeostasis and predisposes ayu (*Plecoglossus altivelis*) to subsequent *Pseudomonas plecoglossicida*-induced infection. Zool. Res..

[22] Yu Y.Y., Huang Z.Y., Kong W.G., Dong F., Zhang X.T., Zhai X., Cheng G.F., Zhan M.T., Cao J.F., Ding L.G. (2022). Teleost swim bladder, an ancient air-filled organ that elicits mucosal immune responses. Cell Discov..

[23] Chen Z.Y., Lei X.Y., Zhang Q.Y. (2012). The antiviral defense mechanisms in mandarin fish induced by DNA vaccination against a rhabdovirus. Vet. Microbiol..

[24] Yao J.Y., Zhang C.S., Yuan X.M., Huang L., Hu D.Y., Yu Z., Yin W.L., Lin L.Y., Pan X.Y., Yang G.L. (2022). Oral vaccination with recombinant *Pichia pastoris* expressing iridovirus major capsid protein elicits protective immunity in Largemouth Bass (*Micropterus salmoides*). Front. Immunol..

[25] Yan N.N., Xu K., Li X.Y., Liu Y.W., Bai Y.C., Zhang X.H., Han B.Q., Chen Z.L., Zhang Z.Y. (2015). Recombinant *Saccharomyces cerevisiae* serves as novel carrier for oral DNA vaccines in *Carassius auratus*. Fish Shellfish Immunol..

[26] Dong Z.R., Mu Q.J., Kong W.G., Qin D.C., Zhou Y., Wang X.Y., Cheng G.F., Luo Y.Z., Ai T.S., Xu Z. (2022). Gut mucosal immune responses and protective efficacy of oral yeast Cyprinid herpesvirus-2 (CyHV-2) vaccine in *Carassius auratus gibelio*. Front. Immunol..

[27] Bøgwald J., Dalmo R.A. (2021). Protection of teleost fish against infectious diseases through oral administration of vaccines: Update 2021. Int. J. Mol. Sci..

[28] Liu Z.X., Wu J., Ma Y.P., Hao L., Liang Z.L., Ma J.Y., Ke H., Li Y.G., Cao J.M. (2020). Protective immunity against CyHV-3 infection via different prime-boost vaccination regimens using CyHV-3 ORF131-based DNA/protein subunit vaccines in carp *Cyprinus carpio* var. Jian. Fish Shellfish Immunol..

[29] Jun J.W., Kang J.W., Giri S.S., Kim S.W., Kim S.G., Kwon J., Lee S.B., Jung W.J., Lee Y.M., Jo S.J. (2022). Preventive effect of starch hydrogel-based oral vaccine against Aeromonas salmonicida infection in rainbow trout (*Oncorhynchus mykiss*). Aquaculture.

[30] Salinas I., Fernández-Montero Á., Ding Y., Sunyer J.O. (2021). Mucosal immunoglobulins of teleost fish: A decade of advances. Dev. Comp. Immunol..

[31] Zhang Y.A., Salinas I., Li J., Parra D., Bjork S., Xu Z., LaPatra S.E., Bartholomew J., Sunyer J.O. (2010). IgT, a primitive immunoglobulin class specialized in mucosal immunity. Nat. Immunol..

[32] Langevin C., Boudinot P., Collet B. (2019). IFN signaling in inflammation and viral infections: New insights from fish models. Viruses.

[33] Stosik M., Tokarz-Deptuła B., Deptuła W. (2021). Type I interferons in ray-finned fish (*Actinopterygii*). Fish Shellfish Immunol..

[34] Xu F., Li M.Y., Chen J. (2020). D-dopachrome tautomerase from Japanese sea bass (*Lateolabrax japonicus*) is a chemokine-like cytokine and functional homolog of macrophage migration inhibitory factor. Zool. Res..

